# Understanding the importance of therapeutic alliance during physiotherapy treatment for musculoskeletal pain in children: a scoping review

**DOI:** 10.3389/fpain.2024.1452771

**Published:** 2024-09-25

**Authors:** Rhiannon Joslin, Eve Allen, Bernie Carter

**Affiliations:** ^1^ School of Health Sciences, Faculty of Environmental and Life Sciences, University of Southampton, Southampton, United Kingdom; ^2^ Women’s and Children’s Department, University Hospitals Sussex, St Richards Hospital, Chichester, United Kingdom; ^3^ Faculty of Health, Social Care and Medicine, Edge Hill University, Ormskirk, United Kingdom

**Keywords:** therapeutic alliance, musculoskeletal pain, child, physiotherapist, child and family centred care, resilience, collaboration, trust

## Abstract

**Introduction:**

Musculoskeletal pain affecting children is common. Rehabilitation and treatment effectiveness can be influenced by multiple individual and contextual factors. The need for more rigorous evaluation of physiotherapy treatment for children's pain, identification of the role of specific techniques, and exploration of the influence of the therapeutic alliance is needed. This scoping review of research aimed to examine: (1) What are the perceptions of children, parents, and physiotherapists about the importance of therapeutic alliance during musculoskeletal pain treatment? (2) What are the key characteristics of therapeutic alliance during a child's musculoskeletal pain treatment from the perspectives of children, parents, and physiotherapists? and (3) What are the perceived impacts of therapeutic alliance (positive and negative) during a child's physiotherapy treatment for musculoskeletal pain?

**Methods:**

The scoping review, based on Arksey and O'Malley's framework and reporting was guided by PRISMA-ScR. The search strategy was based on three concept blocks: (1) Study population: Children (<18 years); (2) Medical condition: Any musculoskeletal pain (acute, chronic primary, chronic secondary); (3) Intervention: Qualitative exploration of experience of physiotherapy treatment delivered by a physiotherapist from the perspective of a child, parent, or physiotherapist. The search (no date limit) was conducted in February 2024 across Medline, AMED and CINAHL.

**Results:**

Following duplicate removal and assessment of eligibility of the initial 236 articles, nine articles were included; of these, only one specifically aimed to explore therapeutic alliance and it was the only paper to directly mention therapeutic alliance. All nine articles presented the child's experience. One overarching theme “Finding resilience within me through therapeutic alliance” and three main themes: “A trusted guide through the ups and the downs of rehabilitation”; “Having a route map”; and “Take me seriously but make it fun” were identified.

**Discussion:**

Therapeutic alliance was considered important by children, parents and physiotherapist and it influenced child and parent perceptions of physiotherapy and overall treatment outcomes. Physiotherapists can foster the children's resilience when experiencing musculoskeletal pain by providing disciplinary expertise, connecting and collaborating with the child by becoming their trusted guide, and co-creating a route map for rehabilitation by helping them to learn about their body, pain and recovery timeline.

## 1 Introduction

Musculoskeletal pain affecting children is common with nearly one in ten seeking primary healthcare each year (1). Children affected by musculoskeletal pain experience physical, social, and emotional impact with most (62%) not expecting a pain-free future (2). Prevalence of chronic musculoskeletal pain is 25.7% (3), prevalence increases in adolescence (4), and pain can continue into adulthood (5–7). Chronic musculoskeletal pain conditions like lower back pain, are a leading cause of global disability (8). Physical interventions are consistently recommended for paediatric musculoskeletal pain despite a paucity of evidence into effectiveness (9–11). When considering rehabilitation more broadly than musculoskeletal pain, multiple individual and contextual factors, including therapeutic alliance, can influence a child's effort in rehabilitation and potentially influence treatment effectiveness (12).

Therapeutic alliance, also termed working alliance is described, within a psychotherapy context, as involving a collaborative relationship, an affective bond between the patient and therapist and agreement on goals and tasks (13–15). Within the field of adult musculoskeletal pain, therapeutic alliance has received increasing research attention with the move towards patient-centred interventions focusing on biopsychosocial approaches (16). A systematic review found that a strong therapeutic alliance appeared to be more effective than traditional physical interventions alone for the treatment of chronic musculoskeletal pain (17). In comparison to adult populations, literature regarding the importance of therapeutic alliance in children is sparse and is primarily focused on the impact of therapeutic alliance on outcomes of children's mental health (18). Children, parents, and health professionals have different roles and responsibilities (19) necessitating triadic therapeutic alliance (20). Factors such as autonomy, known to facilitate therapeutic relationships within adult musculoskeletal care (21), require careful consideration within a paediatric setting to acknowledge the different approaches needed for developmental stages (22). For example, rather than choosing to attend appointments themselves, children are brought by parents or guardians; this automatically changes therapeutic relationships and needs further consideration.

### 1.1 Rationale for the review

While available recommendations advocate child and family centred treatment (10), this is an emerging concept that is poorly defined (23). In their call for action, Eccleston et al. (24) identified the need for more rigorous evaluation of physiotherapy treatment for children's pain, identification of the role of specific techniques, and exploration of the influence of the therapeutic alliance. Understanding the contextual elements of interventions such as the therapeutic alliance, is crucial to establishing treatment effectiveness and improving standards of care.

### 1.2 Core questions

This review is underpinned by three core questions:

1. What are the perceptions of children, parents, and physiotherapists about the importance of therapeutic alliance during musculoskeletal pain treatment and why?
2. What are the key characteristics of therapeutic alliance during a child's musculoskeletal pain treatment from the perspectives of children, parents, and physiotherapists?
3. What are the perceived impacts of therapeutic alliance (positive and negative) during a child's physiotherapy treatment for musculoskeletal pain?

## 2 Methods

A scoping review is a “type of evidence synthesis that has the objective of identifying and mapping relevant evidence that meets pre-determined inclusion criteria regarding the topic, field, context, concept or issue under review”[(25) p4]. A scoping review was most appropriate approach for this research question because it addresses a broad topic (26). The reporting of the review was guided by the Preferred Reporting Items for Systematic Reviews and Meta-analyses Extension for Scoping Reviews (PRISMA-ScR) approach (27). The review protocol has not been published. As this was a scoping review, no ethics approval was required. Our scoping review was based on the five stages proposed by Arksey and O'Malley (2018): identifying the research question; identifying relevant studies; study selection; charting the data; and collating, summarizing and reporting the results. Within this framework we have been meticulous in ensuring that the processes we used reflect the latest guidance on producing a high-quality, rigorous scoping review (e.g., clarity of review questions, concept and context, detailed inclusion criteria, a comprehensive search strategy and transparency and clarity in relation to data extraction) (25). Details are presented within each of the following methods sections.

### 2.1 Inclusion and exclusion criteria

Articles were included if they were:

1. Available in English, as no funding for translation was available.
2. Qualitative studies that explore a child's (aged under 18 years) physiotherapy treatment for musculoskeletal pain, from the perspective of either the child, their parent or physiotherapist.
3. Treatment was delivered by a physiotherapist also known as, physical therapist.

Articles were excluded if they:

1. Solely explored treatment of pain not within the musculoskeletal system. Treatment for headaches, chronic fatigue, or abdominal pain in isolation were not included.

Study designs that used clinical populations where there was a mix of pain locations that included musculoskeletal pain were included in the review.

### 2.2 Information sources and search strategy

For the search strategy, three concept blocks were used within the databases: Medline, The Allied and Complementary Medicine Database (AMED) and Cumulative Index of Nursing and Allied Health Literature (CINAHL).

1. Study population: Children (<18 years).
2. Medical condition: Any musculoskeletal pain (acute, chronic primary or chronic secondary). Musculoskeletal pain defined as pain located within the muscle and skeletal system to include joint and muscle pain, pain post injury e.g., bone fracture, ligament sprain or muscle strain, growth related pain (Osgood Schlatter's, growing pains) and pain secondary to disease such as juvenile idiopathic arthritis. To include mixed populations where musculoskeletal pain included.
3. Intervention: qualitative exploration of experience of physiotherapy treatment delivered by a physiotherapist from the perspective of a child, parent, or physiotherapist.

The search strategy and terms were discussed as a research team and then explored within each database with a librarian familiar with health sciences literature searches. Supplementary File S1 includes the search terms and results per database. The year of publication was not limited. In addition, the key words “child” and “pain” were searched (title and abstract) within the Cochrane database. The search was completed in February 2024.

### 2.3 Selection of sources of evidence (screening and eligibility)

A preliminary pool of 236 articles was identified from the initial searches. Titles and abstracts were reviewed by one reviewer (RJ) and checked by a second (BC). Twelve full text articles were assessed for eligibility by two authors (RJ, BC). Three articles were excluded because the physical intervention was not delivered by a physiotherapist (28–30).

**Figure 1:**
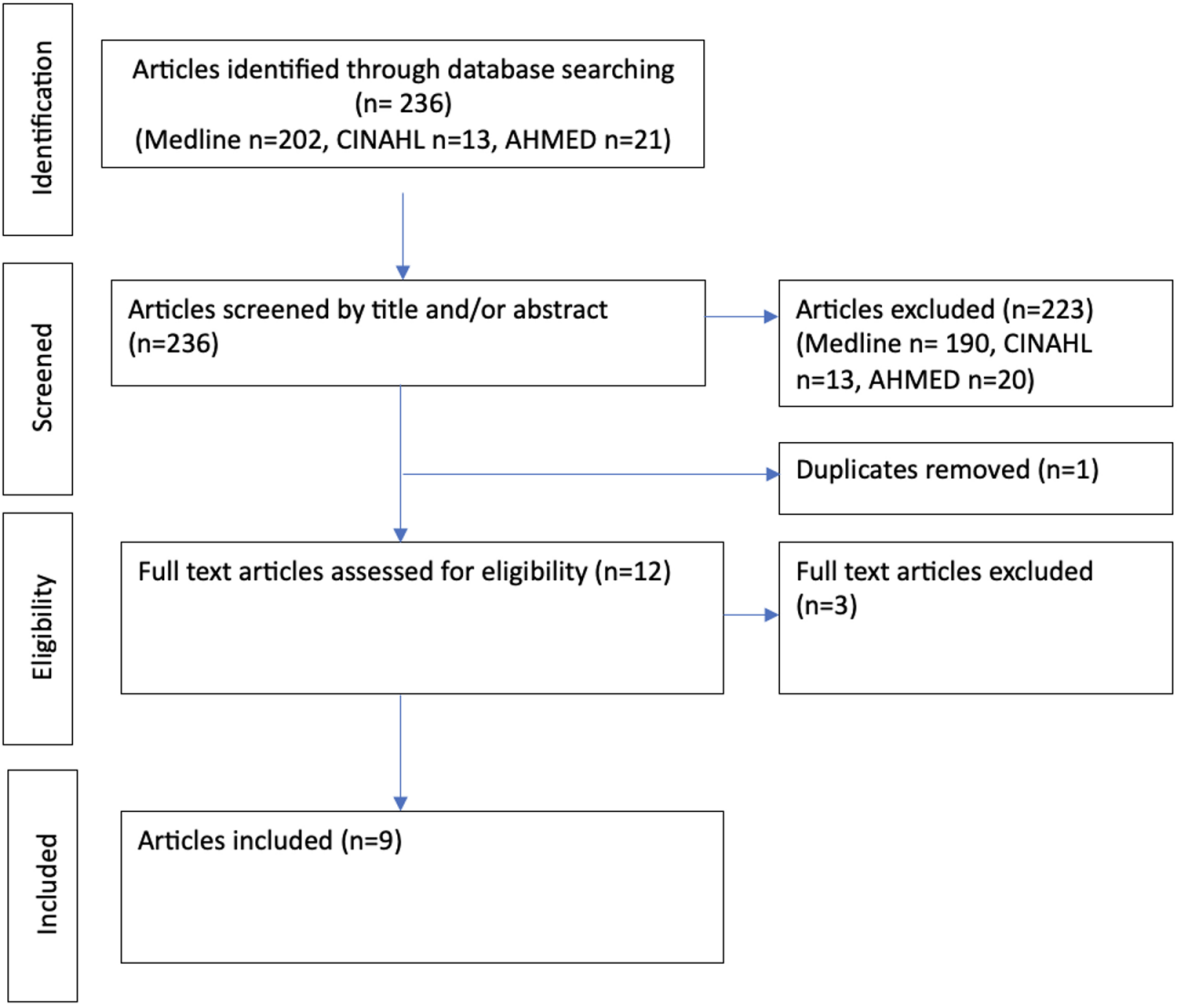
PRISMA flow diagram for scoping review process.

Two articles (31, 32) that included children or young adults outside of our age eligibility criterion were included as both articles provided numbered participant quotes and ages of participants. This allowed us to include findings and quotations specific to participants under 18 years old and exclude findings only evidenced by quotes of participants 18 years or older. Figure 1 summarises the scoping review process.

From the key word search in the Cochrane database, 338 articles were identified, 333 were excluded from the title and the remaining five articles were excluded from reading the abstract as they were quantitative (*n* = 4) (11, 33–35) or the intervention was not physiotherapy (36). To identify possible grey literature, a key word search in the Physiotherapy Evidence Database (PEDro) identified thirty-seven articles. All of these articles were excluded either because they did not address a physiotherapy intervention for children experiencing musculoskeletal pain (*n* = 30) or reported quantitative not qualitative data (*n* = 7).

### 2.4 Critical appraisal

The first two questions (clear aims and appropriate methodology) of the Critical Appraisal Skill Programme (CASP) tool for qualitative studies (37) were used in the initial screening of articles for eligibility. Articles that did not fulfil these first two questions were excluded. Thereafter, the tool was used for quality assessment with the strengths and limitations of the articles presented in the results. Although some authors suggest that critical appraisal is not required or recommended for scoping reviews, we found it a useful process in helping us consider the strengths and limitations of the articles (25).

### 2.5 Data extraction and charting

A data extraction sheet was developed and refined by the research team. Each article was summarised in terms of authorship, publication year, country, setting (inpatient, outpatient, rehabilitation, acute service), discipline/speciality (rheumatology, orthopaedics, child and adolescent mental health, physiotherapy), aims, design, participants, themes, and findings that relate to the scoping review questions.

The initial extraction sheet aligned with the three scoping review questions. Data related to treatment experience was initially extracted from the selected articles (column 1 of the table) to document whether therapeutic alliance was discussed, if so, which components of therapeutic alliance were discussed (column 2) and the perceived impact (column 3). In column two, the components of therapeutic alliance were documented in terms of: (a) relationship with the physiotherapist; (b) agreement of management; (c) agreement on goals; and (d) other components.

Themes and patterns across the dataset were analyzed using the six stages of reflexive thematic analysis (38, 39): (1) data familiarization; (2) systematic data coding; (3) generating themes; (4) review themes, (5) defining, refining, and naming themes; and (6) report writing. Data analysis was completed without the use of software.

The nine articles were coded using the initial extraction sheet by two reviewers independently (RJ, BC). The third author (EA) independently coded a selection (*n* = 4) of the nine articles. All three authors extractions were then amalgamated onto one initial extraction sheet and findings were discussed in a virtual 1-hour meeting with all three authors in terms of themes. Following this meeting two authors (RJ, BC) independently developed three themes. These were compared and discussed by all three authors in a virtual 90 min meeting and an overarching theme was developed alongside the three themes. Themes were finalised and written through stages via email by the three authors. A summary of the data extracted per theme is presented in the Supplementary File S2. The study team (RJ, BC, EA) were multidisciplinary, combined expertise in qualitative methodology, and brought knowledge across pain research, paediatric physiotherapy, children's nursing and children's mental health.

## 3 Results

The results are presented in a narrative format. First, an overview of the articles is presented followed by the final themes.

### 3.1 Overview of the articles

The nine articles (19, 31, 32, 40–45) included in the review present separate studies and are summarised in Table 1.

**Table 1:** Data extraction summary (by author).

Author, yr, country	Aim	Design, methods, participants	Focus for physiotherapy, intervention	Specific mention of therapeutic alliance
Ahlqwist and Sällfors (40) (2012) Sweden	To generate a substantive theory explaining how adolescents succeed in managing their main concerns in daily life.	**Design:** Grounded Theory (within a RCT). **Methods:** Interview. **Participants:** Children and adolescents. ***Children and adolescents:*** (*n* = 14) with low back pain. *Age:* 12–18 years. *Sex:* male (*n* = 6), female (*n* = 8).	**Focus for physiotherapy:** Low back pain. **Intervention:** Individual tailored physiotherapy, & 12-week home exercise programme.	No
Birt et al. (43) (2014) UK	To enhance understanding of factors underlying concordance with multidisciplinary treatment programme for joint hypermobility in children.	**Design:** Qualitative, critical realism (within a RCT). **Methods:** Semi-structured interviews. **Participants:** Children and adolescents, parents. ***Children and adolescents:*** (*n* = 29) with joint hypermobility (only 19 children, 9–17 years interviewed). *Age:* 5–17 yrs. *Sex:* male (*n* = 15), female (*n* = 14). ***Parents:*** (*n* = 32). *Role:* mothers (*n* = 28), fathers (*n* = 4)	**Focus for physiotherapy:** Hypermobility. **Intervention:** Individualised exercise program over 10 weeks in a hospital (outpatient) from physiotherapy and occupational therapy.	No
Blanco-Morales et al. (45) (2020) Spain	To explore current situation of the academic reality in Spain and health status of adolescents, with purpose of improving adolescent health through implementation of a physiotherapy program within the school context.	**Design:** Collaborative action research. **Methods:** Interviews, focus groups, reflexive diaries, field notes. **Participants:** Adolescents, relatives, teachers. ***Adolescents:*** (*n* = 49) with back pain. *Age:* 15–17 years. *Sex:* male (*n* = 29), female (*n* = 20). ***Relatives:*** (*n* = 11). *Sex:* male (*n* = 3), female (*n* = 8). ***Teachers:*** (*n* = 9). *Sex:* male (*n* = 2), female (*n* = 7).	**Focus for physiotherapy:** Back pain. **Intervention:** Classroom based physiotherapy actions over 7-months including: videos, workshops on ergonomics, stretching and massage; stretching exercises and supporting materials.	No
Crom et al. (19) (2020) Netherlands	To explore opinions, perceptions, & preferences of children, parents, & physical therapists regarding therapeutic alliance in paediatric physical therapy in a rehabilitation setting.	**Design:** Phenomenology. **Methods:** Interviews. **Participants:** Children and adolescents, parents, physiotherapists. ***Children and adolescents:*** (*n* = 10) with a range of conditions. *Age:* 3–17 years. *Sex:* not reported. **Parents:** (*n* = 10). *Role:* mothers (*n* = 0), fathers (*n* = 1). ***Physiotherapists:*** (*n* = 10). *Experience:* 3–22 years.	**Focus for physiotherapy:** Factors associated with cerebral palsy, neuromuscular disorders, psychomotor problems and congenital disorder). **Intervention:** Usual physiotherapy care.	Yes
Distanti et al. (42) (2018) USA	To develop an understanding of positive & negative perceptions of rehabilitation and return-to-sport process among adolescent individuals who had not yet returned to sports fol­lowing ACLR.	**Design:** Qualitative. **Methods:** Semi-structured interviews. **Participants:** Adolescents. ***Adolescents:*** (*n* = 10) within 12 months of Anterior Cruciate Ligament surgery. *Age:* 15–18 years. *Sex:* male (*n* = 3), female (*n* = 7).	**Focus for physiotherapy:** Post Anterior Cruciate Ligament reconstruction. **Intervention:** Usual physiotherapy care.	No
Houx et al. (31) (2021) France	To improve understanding of the pain experience of children and young adults (CYA) with cerebral palsy during physiotherapy sessions.	**Design:** Qualitative. **Methods:** Focus groups. **Participants:** Children and adolescents. ***Children and adolescents:*** (*n* = 18) with cerebral palsy. *Age:* 8–20 years. *Sex:* male (*n* = 8), female (*n* = 10).	**Focus for physiotherapy:** Cerebral palsy (67% GMFCS levels III-IV). **Intervention:** Usual care. Mean number of physiotherapy sessions per week was 3 (SD 0.79).	No
Kuenze et al. (41) (2022) USA	To examine perceptions of information sharing & interpersonal communication among adolescent patients recovering from ACLR, one of their parents, and physical therapists who provided rehabilitative care for patients with ACLR.	**Design:** Qualitative (within clinical outcomes trial). **Methods:** Semi structured interviews. **Participants:** Adolescents, parents, physiotherapists. ***Adolescents:*** (*n* = 9) following Anterior Cruciate Ligament reconstruction surgery. *Age:* 14–18 years. *Sex:* male (*n* = 2), female (*n* = 7). ***Parents:*** (*n* = 9). *Role:* mothers (*n* = 7), fathers (*n* = 2). ***Physical therapists:*** (*n* = 9). *Experience:* 2–26 years. *Sex:* male (*n* = 3), female (*n* = 6).	**Focus for physiotherapy:** Post Anterior Cruciate Ligament reconstruction. **Intervention:** Usual care.	No
Paterno et al. (32) (2019) USA	To identify rehabilitation factors adolescent & young adult patients & their parents perceive as having strongly impacted their outcomes after ACLR.	**Design:** Interpretive phenomenology. **Methods:** Semi-structured interviews **Participants:** Adolescents, parents. ***Adolescents****:* (*n* = 10) following Anterior Cruciate Ligament reconstruction (only 9 interviewed). *Age:* 12–21 years. *Sex:* male (*n* = 6), female (*n* = 4). ***Parents:*** (*n* = 10) (only 9 interviewed). *Role:* mother (*n* = 9), father (*n* = 1)	**Focus for physiotherapy:** Post Anterior Cruciate Ligament reconstruction. **Intervention:** Usual care	No
Williams et al. (44) (2015) UK	To explore factors that influenced participants’ acceptability & perception of the trial & interventions, issues influencing exercise adherence & appropriateness of the chosen outcome measurements.	**Design:** Qualitative (part of a feasibility study) **Methods:** Interview **Participants:** Adolescents, parents, physiotherapists. ***Adolescents:*** (*n* = 6) with a diagnosis of idiopathic adolescent scoliosis (AIS). *Age:* 10–16 years. *Sex:* male (*n* = 0), female (*n* = 6). ***Parents:*** *n* = 6 *Role:* mothers (*n* = 4), fathers (*n* = 2). ***Physiotherapists:*** (*n* = 4) *Experience*: experienced in providing exercise intervention for AIS (*n* = 2), less experienced (*n* = 2).	**Focus for physiotherapy:** Idiopathic scoliosis **Intervention:** Six to nine sessions of education, advice & a programme of Scoliosis Specific Exercises, supported by daily home exercise programme.	No

#### 3.1.1 Dates of publication and country

Despite no limit on date of publication, included articles were from a ten year range from 2012 (40) to 2022 (41).

Articles represented studies internationally in the USA (32, 41, 42), United Kingdom (43, 44), France (31), Netherlands (19), Spain (45), and Sweden (40).

#### 3.1.2 Study design

Of the nine articles, four were qualitative sub-studies that explored experiences of a physical interventions qualitatively as part of a larger programme of research: of these programmes two were randomised controlled trials (40, 43) and the other two were feasibility (44) and collaborative action research (45) studies. The remaining five articles explored experiences of physical interventions during usual physiotherapy care in routine clinical practice (19, 31, 32, 41, 42).

Only one article (19) specifically aimed to explore therapeutic alliance within paediatric physiotherapy and it was the only paper to directly mention therapeutic alliance. The remaining articles had broader aims relating to understanding perceptions (42), acceptability (44) and implementation (45) of physiotherapy, with articles focusing on understanding self-management (40), concordance (42), communication and information sharing (41), and factors influencing outcomes (32).

#### 3.1.3 Focus on experience

All nine articles presented the child's experience. Four articles explored the experiences of children, parents, and physiotherapists (19, 41, 44, 45). Three articles solely explored the child's experience (31, 40, 42), two articles explored the experiences of children and parents (32, 43). The nine articles resulted in the viewpoints of 146 young people (73 female, 63 male, 10 not reported), 79 parents (66 female and 13 male) and 32 physiotherapists (6 female, 3 male, 23 not reported) being represented.

#### 3.1.4 Population of interest

Of the nine articles, eight addressed specific populations: ACL injuries (*n* = 3) (32, 41, 42); back pain (*n* = 2) (40, 45) of which one which was specific to low back pain (40); hypermobility (*n* = 1) (43); idiopathic scoliosis (*n* = 1) (44); and cerebral palsy (*n* = 1) (31). One study addressed a heterogenous population of children receiving rehabilitation including children with cerebral palsy and orthopaedic problems (19). This meant that seven articles (32, 40–45) involved conditions that typically require short-term physiotherapy management (periods of rehabilitation that have an endpoint) and two articles involved conditions that typically require long-term physiotherapy management (potentially lifelong) (19, 31). Most of the articles included young people considered to be within the adolescent age range of 10–18 years old, although Crom et al. (19) included a younger age (range 3–17 years old). Houx et al. (31) included 3 (22%) of 18 participants who were aged 19 and 20 years old; however, the mean age across all participants was 13.17 years. Paterno et al. (32) included 2 (20%) of 10 participants who were 18 and 21 years old; however, the mean age across all participants was 16.9 years.

#### 3.1.5 Strengths and limitations of literature

Recruitment strategies were appropriate, with three articles detailing how they used purposive (31, 40, 43) and one using theoretical sampling methods (45). Apart from Houx et al. (31), all other articles clearly outlined ethics approval and informed consent procedures.

All the articles explained how qualitative data was collected in a way that addressed the research issue using open interviews (40), semi-structured interviews (19, 32, 41–45) and focus groups (31). While most studies sought child, parent, and physiotherapist perspectives in separate interviews (19, 31, 40–42, 44, 45), Paterno et al. (32) and Birt et al. (43) interviewed the child and parent together. Ahlqwist and Sallfors (40) and Blanco-Morales et al. (45) reported the main author delivered the intervention although it was unclear who interviewed the participants. The relationship between the researchers, interviewers and participants were outlined in the remaining studies and demonstrated the interviewer was independent to the child's treatment.

Some studies were strengthened by developing the interview schedule with relevant stakeholders (32, 44, 45), pilot testing the interview (19, 41), giving the participant a choice on where they wanted to be interviewed (44), and including the interview guide (19, 41). All the articles reported audio recording and transcribing verbatim. Methods of data analysis varied and included phenomenological analysis (19, 32, 44), thematic analysis (31, 41–43), axial coding (45) and using classical grounded theory (40). Data analysis was strengthened by using multiple coders (19, 31), developing themes with multiple researchers (40, 41, 44), triangulation of multiple sources of data (19), reflecting on individual biases and experiences during coding (32) and member checking (19, 43).

### 3.2 Themes

Finding resilience within me through therapeutic alliance, was an overarching theme across the three main themes, some with subthemes, that were generated: a trusted guide through the ups and the downs of rehabilitation; having a route map; and take me seriously but make it fun.

#### 3.2.1 Overarching theme: finding resilience within me through therapeutic alliance

This overarching theme ultimately highlights the importance of personalising physiotherapy and children taking an active role (40).

Physiotherapy treatment offered a vehicle for children experiencing musculoskeletal pain to find resilience within themselves, providing hope for the future. Children described how physiotherapy provided them with an opportunity to regain some control over their body (40, 44, 45) and life (40, 44), the confidence to take steps to influence their own situation (32, 40, 41, 43, 44) and self-manage (32, 40, 43–45). Parents valued observing their child make their own choices, grow as a person, and self-manage (44) and parents needed to feel involved (19).

Children who were athletes were more likely to see physiotherapy treatment as positive, if they viewed the process as “overcoming adversity” (42), an experience that made them stronger as a person (42). The ability to overcome challenges for their “own good” was reported by most of the children who required stretches to manage cerebral palsy (31). Knowing what to expect (41, 42) and establishing a strategy that worked, created a sense of autonomy, that resulted in positive outcomes such as feeling able to prevent deterioration (43, 44), manage their long-term condition (44), reduce pain (40, 44, 45) and, improve their function (42), health, and wellbeing (45).

Children found resilience in themselves by developing their own personal “tool kit”. On a superficial level, “tools” included posture awareness (44, 45), relaxation (45), stretches (31, 45), exercises (40, 43, 44) and distraction (40); however, it was the context of how interventions were delivered that mattered.

Across all nine articles, therapeutic alliance offered critical and fundamental tools for change, these key findings are now described across the three main themes.

#### 3.2.2 Theme 1: a trusted guide through the ups and downs

When children faced the uncertainty of physiotherapy treatment, they and their parents, highlighted the importance of having a physiotherapist they trusted to guide them through their journey. Trust within the triadic relationship (child-parent-physiotherapist) and guidance from the physiotherapist appeared key to therapeutic alliance.

##### 3.2.2.1 Trust

The crucial importance of trust was acknowledged by all stakeholders (children, parents, and physiotherapists) (19, 40, 42). Trust was considered a prerequisite to treatment that allowed a child to feel safe (19). Trust was reported when physiotherapists were perceived to be listening, taking problems seriously, and showing understanding (40). Two types of trust were described: trust in relational skills and trust in technical skills (19). Physiotherapists associated being a good professional with technical skills whereas, children and parents placed more importance on relational trust (19). Trust was fragile and there were examples where young people reported a sense of betrayal (31) or poor communication that undermined decisions and negatively impacted trust (41).

Children and parents identified a trusting relationship with the physiotherapist was pivotal during negotiations about treatment goals (19), a positive recovery factor (42), and played a key role in the perception of family-centered care (19).

##### 3.2.2.2 Being a guide

Children wanted physiotherapists to guide them throughout the rehabilitation process (32) and to inspire them to do the things they used to and new things in the future (40). Children wanted their physiotherapist to supervise exercises and treatment (32, 40, 43, 44) so they felt able to challenge and expose themselves to situations they had previously avoided (40) and; motivate (32, 40–42, 44) them to stay on the right track (32) especially when there were psychological barriers (42) and low points in rehabilitation (32, 41). They described a successful (physiotherapy) guide as being someone who appeared to genuinely want to help (40, 44) and who was kind (32), warm (40), open (19, 40), and empathetic (19). Physiotherapy guides were able to incorporate the holistic needs of children and parents into their thinking and reasoning (19, 44). Successful guides had been trained (40) and brought expertise (44, 45) and knowledge (of the child's health condition and treatment) (19, 45) into the alliance. They were willing to be honest and transparent in their communication (19). Although transparency was sought, some parents reported they did not share difficulties experienced during appointments with their physiotherapist (43) and when physiotherapists noticed problems with the therapeutic relationship they neither reflected on nor discussed these openly with children or parents (19). Physiotherapists were recognised as being an “important voice” in the coordination of different stakeholders, filling gaps and maintaining consistent expectations to successfully progress rehabilitation (32).

Having a consistent physiotherapist with them on their rehabilitation journey meant children did not feel alone, perceiving the journey as a “joint effort” (40). Physiotherapists could boost a child's confidence (32), supporting them to push past invisible boundaries, face fears (32) and navigate uncertainties (40). With this support, over time, children found their own solutions, mobilised their own resources, and took an active role to find their own path (40).

Therapeutic relationships had the potential to both positively and negatively affect perceptions of the rehabilitation process (41) and was a key driver of outcome (32). Not having guidance and attention from the physiotherapist was a factor identified by children as negatively impacting recovery (42). In combination with trust, guidance from a physiotherapist throughout rehabilitation appeared to give children control of their musculoskeletal pain and its consequences (40, 45).

#### 3.2.3 Theme 2: having a route map

An important aspect of the therapeutic alliance was the way in which physiotherapists provided children with information—a route map—for their treatment and/or ongoing self-care. Information which built children/young people's knowledge was described as powerful (45) and beneficial (42), as it could promote more positive (42) experiences.

##### 3.2.3.1 A realistic and understandable route

Despite physiotherapists being perceived as having disciplinary expertise there was recognition that a three-way flow of information between the key stakeholders (child-parent-physiotherapist) was essential (19) to inform tailored interventions and make “things work” (19). However, despite a wealth of expertise, physiotherapists may lack expertise in some areas which in turn may impact on the experience of the child/young person (31).

Although other sources of information such as social media or the internet were acknowledged, these were seen to be adult-centric (40), offered worst case (44) or worrying (40) scenarios and failed to provide the specificity, knowledge and expertise available from a physiotherapist. Expert information could reduce the emotional impact of injury through reducing fear (40, 41), anxiety, isolation and uncertainty (41). Parents talked of the importance of physiotherapists understanding the emotional impact of a diagnosis on their child (19).

The notion of a route map was evident in how information was core to managing expectations in terms of timelines (32, 41) about recovery. Having a clearer timeline helped children understand that recovery was not necessarily going to be easy (41) or linear (42).

Context-relevant and tailored information supported overall understanding (32, 40), understanding of causes (44), motivation (32, 41) and shifted focus away from symptoms (40) towards more nuanced understanding. Such understandings included how to proceed (40) and how to transfer knowledge to other settings (45).

Information helped children to build a sense of control (42) and encouraging children (40) and parents (19) to accept a greater sense of responsibility for self-care and self-management (45). It also informed how they could manage future exacerbations of symptoms (43).

The absence of suitable information resources was noted (41).

##### 3.2.3.2 An agreed sense of direction

Goals were identified as being useful (44) with an emphasis on tailored, specific goals (42) with co-ordination by the physiotherapist being identified as key (32). Collaborative, self-determined goals were considered the best approach (19, 42) with negotiation occurring, as needed, between the child, parents and physiotherapist. However, although collaborative goal setting was considered ideal, factors such as negativity about the treatment or a child being unwilling or unable to express their ideas limited collaboration (19). Collaboration or consensus on goals is not inevitable and lack of agreement can rupture the therapeutic alliance (19) and shared goals do not necessarily guarantee adherence to a programme (43). Other factors such as a lack of understanding on the part of the physiotherapist can limit collaboration (31).

Clear and tailored goals can shift the focus from the impairment (44) or symptoms (40) to something that encompasses more than just physical markers of improvement (42) and improvements in motor skills (19) to a more balanced holistic approach that acknowledges the whole person (19) and which uses relevant motivators (42). Timelines embedded with realistic expectations are core to goal planning (32, 41).

#### 3.2.4 Theme 3: take me seriously but make it fun

Fun was a term consistently used by children in relation to all aspects of therapeutic alliance. Children highlighted the importance of enjoyment during physiotherapy treatment (31, 40, 43–45) and goal setting (40) and how physiotherapists who were interesting and fun created positive relationships (44). Creating a safe, relaxed atmosphere where the physiotherapist smiled and there was room for jokes was important but so was the need to be respectful and take the young person and their concerns seriously (19). Children and parents wanted physiotherapists to support children's emotional needs because they felt no one talked about the ‘head game’ and mental toll children faced during treatment (32, 41). During rehabilitation periods associated with frustration and depression, children associated talking to their physiotherapist about life (music, sports etc.) with having a “good time” (41).

Physiotherapy treatment, such as exercises, could be enjoyable (43, 44) but could also be monotonous (43), boring (31), tiring (31) and difficult to fit round normal routines (43). When physiotherapy treatment was perceived as a burden, and generic (not personalised) (42) it was not prioritised by children (43). In addition, negative consequences, such as pain, led to a general dislike of physiotherapy (31). Fun/enjoyment is individual and relates to a child's developmental and cognitive stage. For younger children, a home exercise program was successfully maintained when parents or a whole family got involved and turned exercises into a competition (43). However, when older children were expected to complete exercises alone, they wanted to see a link between their treatment and meaningful goals such as returning to sport (42) or a valued activity (43).

Fun encompasses the whole child, and the people and world around them. Social and environmental aspects of physiotherapy management were reported to have a large impact on the perceptions of the rehabilitation experience (32), with positive factors including being greeted by name, having a range of equipment and available space (32). Conversely, negative social impacts such as social comparison (42) and parental anxiety (41) were described. Parents and children expressed the importance of considering a broader range of goals related to self-management, happiness, or body confidence (44). Some physiotherapists whose focus was on setting goals related to physical factors, struggled when children could function physically but still experienced pain (44).

When young people were enjoying themselves, they reported increased energy, reduced pain and a general improving in wellbeing (40). This experience was linked to the young person being more motivated to keep going (40), higher exercise adherence (43) and more likelihood of seeing their recovery positively (42).

## 4 Discussion

This scoping review explored the concept of therapeutic alliance (13) within physiotherapy treatment for musculoskeletal pain in children. The review identified that the therapeutic alliance was considered important by all stakeholders (child, parent and physiotherapist). Evidence suggested therapeutic alliance influenced child and parent perceptions of physiotherapy and overall outcomes of treatment. Fostering resilience of children experiencing musculoskeletal pain through the therapeutic alliance was a key finding and overarching theme. The described characteristics of therapeutic alliance, from a range of critically appraised articles, has provided new knowledge to inform clinical practice.

In considering the characteristics of the articles included in the review we note that only one of the nine articles (19) specially addressed therapeutic alliance; there may be several reasons for this. Therapeutic alliance may not yet be either a concept of interest or a research priority within paediatric physiotherapy musculoskeletal pain practice. Alternatively, funding may not be available to support therapeutic alliance studies within paediatric populations. The review suggests the need for good quality primary rather than a secondary outcome studies addressing therapeutic alliance in paediatric physiotherapy musculoskeletal pain practice. Further, within the review the focus of most articles was on short-term, end-point oriented (e.g., ACL) rehabilitation (32, 40–45) whereas, arguably, more substantive therapeutic outcomes may accrue if future research focuses on longer-term rehabilitation (e.g., children with cerebral palsy) (31). Considering the intrinsic triadic nature of paediatric physiotherapy practice, it was perhaps surprising that only four articles (19, 41, 44, 45) addressed the perspectives of children, parents and physiotherapists. This would seem essential for therapeutic alliance research particularly for younger children, albeit that the focus might shift to a more dyadic, therapeutic alliance focus for older adolescents with long-term conditions as a means of enhancing independence from parents.

There is not a universal definition of resilience, but it is described as a dynamic and contextual process in response to adversity or challenges (46). Children from the current scoping review highlighted the dynamic nature of resilience and reported that low points of rehabilitation could be overcome (42) and physiotherapy provided an opportunity to regain some control over their body (40, 44, 45) and life (40, 44). This appeared to facilitate self-management (32, 40, 43–45) a key priority for reducing the impact of paediatric musculoskeletal conditions (47). Previous literature in the field of children's chronic pain, has explored the concept of flourishing (positive outcomes as a result or despite chronic pain) (48–50) with children noting that flourishing can lead to ‘becoming a better version of myself’ (48). This ability to overcome adversity and flourish, has been echoed by Looman et al. (51) whose timeline work explored children's perceptions of resilience noting that children who had experienced an adverse event or mental health challenge drew rebound points (a low point in their life followed by a sharp improvements). Interestingly, when Joslin et al. (52) explored children's treatment experiences of chronic musculoskeletal pain using a similar timeline method, children drew these same rebound points when they overcame low points of rehabilitation, and these appeared pivotal to overall recovery. A consistent feature underlying resilience from the current scoping review and previous literature, is the importance of positive relationships (51) with health professionals (48, 52).

Placing resilience as an overarching theme supports the work from Masten (53), contributing to the premise that physiotherapists can place “ordinary magic” into the therapeutic alliance. “Ordinary magic” is considered an attribute inherent in all people; however, physiotherapists are well-placed during musculoskeletal rehabilitation to provide personal strength to children through positive experiences and/or support at stressful or challenging periods. It has also been reported by Gmuca et al. (54) that children experiencing chronic musculoskeletal pain have low to moderate levels of resilience and lower levels of resilience are associated with poorer quality of life, greater functional disability, and higher pain intensity. This supports the need to focus on building resilience in this population and the potential for resilience to influence health outcomes.

From the current scoping review, one way for physiotherapists to engage actively in the therapeutic alliance would be to facilitate the growth of a child's toolbox of coping mechanisms; tools that not only support the physical health of the child, but also recognises their emotional and social needs (19, 40, 44). A coping skills toolbox could enhance the therapeutic alliance, enabling children to ‘bounce back’ or ‘bounce forward’ (53), face their fears and challenge uncertainty (32, 40). In this scoping review, key components of the therapeutic alliance that appeared fundamental to children establishing resilience include the intrinsic need to feel safe (19) and trust the physiotherapist (19, 32, 40). Trust has been identified as being central to a child's therapeutic relationship with nurses (55) and a key finding in this scoping review was that children wanted to trust their physiotherapist to guide them through rehabilitation. These aspects align with a strength-based perspective (56) of engagement in which the child's strengths are identified and built enabling them to be supported by the adults surrounding them (57). The current scoping review identified that trust could be broken (31, 41) and various factors such as the personal qualities of the physiotherapist and their relational skills were important (19); relational skills have been noted in other studies as being factors that can facilitate or hinder trust (58). Like resilience, the concept of trust is a process (59) and, if broken, it can be re-established (58). Physiotherapists need to acknowledge factors such as a child's age which may influence trust (55, 59), monitor the therapuetic relationship, discuss its importance, and address potential fractures with children and parents (19). Overall, the bonds which promote trust (13) are core to therapeutic alliance and involve striving “to view the world and the concrete situation through the eyes of the children and meet them on their own terms” [(40) p2].

To enrich the therapeutic alliance, children in the current scoping review needed to find their route map, they required context-relevant and tailored information to provide better understanding (40, 44, 46). Physiotherapists, parents, and children could then be on the same page, working collaboratively to keep on track, monitor and maintain motivation (19, 42). A scoping review by Holt et al. (60) aimed to identify barriers and facilitators of exercise adherence in youth with musculoskeletal studies. Unlike the current scoping review, Holt et al. (60) included quantitative and qualitative studies and the person delivering the exercise intervention did not need to be a physiotherapist. Holt et al. (60) also found that components of therapeutic alliance boosted exercise adherence, such as education, instruction and demonstration of behaviour, reinforcement (feedback and monitoring) and social support; however, there was a paucity of evidence on the value of goal setting.

Evidence in the current scoping review suggested that physiotherapy goals focus on a child's physical needs (19) whereas parents and children sought broader, holistic goals (19, 44) related to self-management, happiness, and meaningful engagement with life (44). These goal-related disparities between the key players (child-parent-physiotherapist) in the therapeutic alliance triad have the potential to create tensions. For example, a physiotherapist's focus on the musculoskeletal system may lead them down a more focused biomedical route that is at odds with the more holistic desires of the child. What may add to this disparity is the desire for children to have treatment and goals that are fun (30, 31, 40, 43–45) and focused on enjoyable activities (43). Fun and enjoyment are contextual and require physiotherapists to have a holistic understanding of the child and family, shifting their focus from body structure to meaningful participation with life. This approach goes beyond the biopsychosocial approach (61) and aligns with ecological-enactive approaches presented for adult chronic musculoskeletal pain (62). The ecological-enactive framework presented by Vaz et al. (62) would involve the child's individual needs being the central focus, the physiotherapist validating a child's lived experience, seeking to understand their and their parents’ beliefs and perceptions, control contextual factors to create a safe environment to perform feared activities, and create opportunities for action based on self-identified enjoyable goals (62). Acknowledging the holistic needs of the child and family and uniqueness of each family web (63) ultimately support best health outcomes (19, 32, 40).

Across the scoping review themes, a child's need for safety (19) and belonging in terms of creating and maintaining interpersonal and social bonds with their physiotherapist (19, 31, 32, 40–42, 44, 45), and exercises and activities that linked to meaningful goals (19, 32, 42–44) were evident and overlapped with human motivation theories such as the Self-Determination Theory (64) and Maslow's Hierarchy of Needs (65). This raises the question whether addressing these basic needs facilitates behavioural change during physiotherapy. The findings of this scoping review in relation to family-centred care (19) resonate with literature summarising person-centred care within adult physiotherapy (66) that values the importance of support, individualising treatment, providing education, continuous communication, and patient defined goals (66). Such an approach requires physiotherapists to have the confidence, knowledge, and social skills to work in this more equitable way (66). Ultimately, this scoping review supports the need to personalise physiotherapy treatment for children experiencing musculoskeletal pain to support child and family centred care.

It is important to acknowledge limitations of this scoping review. Firstly, compared to systematic reviews, scoping reviews have methodological limitations and are at risk of bias as the process of review is less rigorous and the more opportunistic surveying of the literature can be limiting (67). In turn this means findings may be significantly limited in terms of providing “concrete guidance” [(68) p3]. Secondly, the paucity of evidence in this field meant findings were reliant on nine articles with diversity in age range, delivery of treatment and underlying condition; key populations such as children with inflammatory joint disease were not represented. In addition, some differences were noted in the experiences of therapeutic alliance when a child had a permanent long-term condition in comparison to acute musculoskeletal injuries; however, with limited studies this requires further exploration. Thirdly, all the studies explored in-person physical interventions, further research could explore how telehealth delivery influences development of therapeutic alliance, something that has been explored in adult populations (69). In terms of further research, key elements of therapeutic alliance identified as important in this scoping review such as trust continue to be neglected areas of research (70–72). It would be important to understand the pragmatic implications of how to foster therapeutic alliance within a model of child and family centred care, and the potential implications on the treatment experience, and health outcomes.

## 5 Conclusion

Therapeutic alliance was identified as important by all stakeholders and appeared to influence the experience and outcomes for children treated for musculoskeletal pain. In terms of key characteristics evident in the review, core to therapeutic alliance is a collaborative relationship, an affective bond between the patient and therapist and agreement on goals and tasks (13–15). By acknowledging the importance of therapeutic alliance and how this links with a child's resilience, we can further understand how children can maintain a positive experience with their physiotherapist.

Physiotherapists need to be a child's “trusted guide” providing disciplinary expertise, support and collaboratively creating a route map; the basis of this is understanding a child's basic needs, such as feeling safe and not being judged. They also need to personally connect with children through active listening and getting to know them; by doing this they can engage and support children to learn about their body, pain, and recovery timelines. If physiotherapists can gain trust and successfully support and guide children and their parents, children have the opportunity to draw on their own resilience, equip themselves with long-term management skills and problem solve. Without these fundamental components to the therapeutic alliance, pain management will be sub-optimal, trust in the physiotherapist either never builds or diminishes, and hope for rehabilitation and wellbeing can be lost. It is crucial that therapeutic alliance is central to physiotherapy practice. Further research needs to explore how therapeutic alliance can be fostered to potentially improve outcomes for children and their families.

## Data Availability

The original contributions presented in the study are included in the article/**Supplementary Material**, further inquiries can be directed to the corresponding authors.

## Supplementary material

Supplementary File S1 Search Strategy and results.

Supplementary File S2 Data Extraction Table.
