# Supplementary material for: Understanding the importance of therapeutic alliance during physiotherapy treatment for musculoskeletal pain in children: a scoping review
Source: Front Pain Res (Lausanne). 2024 Sep 25;5:1452771. doi: 10.3389/fpain.2024.1452771 (PMC11461466; doi:10.3389/fpain.2024.1452771)
Supplement: Supplementary File S1 — Search Strategy and results. [file Datasheet1.pdf]

Search Strategy and Results

Database CINAHL

Search terms:

1. (MH "Physical Therapy") OR (MH "Pediatric Physical Therapy") OR (MH "Physical Therapy Practice") OR (MH "Physical Therapy Practice, Evidence-Based") OR (MH "Home Physical Therapy") OR (MM "Rehabilitation+") OR (MM "Therapeutic Exercise+") OR (MM "Exercise+") OR (MM "Group Exercise")

AND

2. (MM "Musculoskeletal Abnormalities+") OR (MM "Musculoskeletal Pain") OR (MM "Musculoskeletal Diseases+")

AND

3. (MM "Qualitative Studies+")

AND

Limiters - Age Groups: Infant, Newborn: birth-1 month, Infant: 1-23 months, Child, Preschool: 2-5 years, Child: 6-12 years, Adolescent: 13-18 years
= 3 (none relevant)

Limiters - Age Groups: All Child
= 13 (none relevant)

(MM "Child+") OR (MM "Adolescence+") OR (MM "Athletes, High School")
= 0

MEDLINE

1. (MM "Physical Therapists") OR (MM "Physical Therapy Specialty") OR (MM "Physical Therapy Modalities+") OR (MM "Physical Therapy Department, Hospital") OR (MM "Aquatic Therapy") OR (MM "Exercise Therapy+") OR (MM "Physical and Rehabilitation Medicine+") OR (MM "Exercise+") OR (MM "Massage+") OR "physical therapy"

AND

2. "Musculoskeletal Pain+") OR "Musculoskeletal Pain" OR (MM "Musculoskeletal Development+") OR (MM "Neck Pain") OR (MM "Musculoskeletal Abnormalities+") OR (MM "Musculoskeletal Diseases+") OR (MM "Flank Pain") OR (MM "Back Pain+") OR (MM "Chronic Pain") OR (MM "Acute Pain") OR (MM "Pain Management") OR (MM "Shoulder Pain") OR (MM "Chest Pain+") OR (MM "Low Back Pain") OR (MH "Patellofemoral Pain Syndrome") OR (MM "Complex Regional Pain Syndromes+")

AND

3. (MM "Qualitative Research+") OR (MM "Focus Groups") OR "qualitative"

AND

4. (MM "Child+") OR "child" OR (MM "Adolescent") OR "adolescents" OR (MM "Youth Sports") OR "youth"

Year of publication NOT limited – 1997-current
=75

Relevant

4. Ahlqwist and Sällfors C. 2012. Experiences of low back pain in adolescents in relation to physiotherapy intervention. *Int J Qual Stud Health Well-being*, 7:1 15471, DOI:10.3402/qhw.v7i0.15471

5. Birt et al. 2014. Adherence to home physiotherapy treatment in Children and Young People with Joint Hypermobility: A Qualitative Report of Family Perspectives on Acceptability and Efficacy. *Musculoskeletal care,* 12:56-61
6. Palmer et al. 2016. Physiotherapy management of joint hypermobility syndrome- a focus group study of patient and health professional perspectives. Physiotherapy. Mar;102(1):93-102. doi: 10.1016/j.physio.2015.05.001.
7. Sims-Gould et al. 2018. "I just want to get better": experiences of children and **youth** with juvenile idiopathic arthritis in a home-based exercise intervention. *Pediatr Rheumatol,l* 16, 59 (2018). https://doi.org/10.1186/s12969-018-0273-6
8. Sieczkowska et al. 2022. A home-based exercise program during COVID-19 pandemic: Perceptions and acceptability of juvenile systemic lupus erythematosus and juvenile idiopathic arthritis adolescents. *Lupus,* Apr;31(4):443-456. doi:10.1177/09612033221083273.
9. Astley et al. 2021. Home-based exercise program for adolescents with juvenile dermatomyositis quarantined during COVID-19 pandemic: a mixed methods study. *Pediatr Rheumatol Online J*, Nov 13;19(1):159. doi: 10.1186/s12969-021-00646-7.
10. Blanco-Morales et al. (2020) Implementation of a Classroom Program of Physiotherapy among Spanish Adolescents with Back **Pain**: A Collaborative Study. *Int J Environ Res Public Health.* 2020 Jul 3;17(13):4806. doi: 10.3390/ijerph17134806.
11. Williams et al. 2015. Active Treatment for Idiopathic Adolescent Scoliosis (ACTIvATeS): a feasibility study. Health Technol Assess. Jul;19(55):1-242. doi: 10.3310/hta19550.

Excluded from title

1. Population adults.
   Battista et al. 2022; Garzonio et al. 2022; Barber et al. 2021; Winzenberg et al. 2008;
2. Population not MSK
   Setchell et al. 2022; Mengshoel et al. 2020; Senesac et al. 2020; Lee et al. 2020; Warnink-Kavelaars et al. 2019; Skogvold et al. 2019; Thille et al. 2018; Ezzat et al. 2018; Barnard et al. 2018; Setchell et al. 2018; Hind et al. 2017; Brigden et al. 2016; Boulay et al. 2015; de Carvalho et al. 2015; Gaab and Steinhorn 2015; Durlacher et al. 2015; Yeh ML et al. 2013; Haumont et al. 2011; Gilmore et al. 2010;
3. Not a qualitative study – commentary
   Commentary Kenyon et al. 2019; McHugh R; Allen M, 2018;
   Not qual- Swierkosz and Nowak 2015; Niesluchowski et al. 1999; Engelbert et al. 1998; Hodgkinson et al. 1997;
   Protocol- Evans et al. 2011;

Not experience of PT:

1. MDT rehab - Shulman et al. 2023; Toupin-April et al. 2023; Ruskin et al. 2023; Kashikar-Zuck et al. 2016; van Wijck et al. 2015;
2. Lived experience general - Wall et al. 2023 (LBP athlete); Salvo et al 2023; McLaughlin et al. 2022; Harry et al. 2022; Gremillion et al. 2022; Molina-Garcia et al. 2021; Terry et al. 2015; Tong et al. 2012;
3. Consent validity questionnaire/outcome measure- Alamrani et al, 2023; Hoglund et al. 2023; Alamrani et al. 2021; Flodén et al 2019; Heyworth et al. 2018;
4. Physical activity/exercise - Tucker et al. 2023; Aviram et al. 2022; Acer Kasman et al. 2021; Bekkering et al. 2012;
5. Radiography- Miller et al. 2022; Ladd et al. 2021
6. Development of decision aid/screening tool/- Ho et al. 2022; Mesaroli et al. 2021;
7. Back pain information- Santos et al. 2022;
8. Epidioiomolgy - Palacios-Ceña et al. 2021;
9. Behavioural model- Talty et al. 2020;

10. Animal therapy - Charry-Sánchez et al. 2018
11. Other Rx – Boardman et al. 2011; Jayawardena et al. 2011; Antoniazzi et al. 2000;
12. Fitness instructor- Hutzal et al. 2009;
13. Orthoses- Näslund et al. 2003

Excluded abstract

- Mixed HCP not just PT- Alamrani S; et al 2024,

New search:

Additional search in Medline excluding MSK to establish studies which may have mixed populations including children with MSK pain.

1. (MM "Physical Therapy Specialty") OR (MM "Physical Therapists") OR (MM "Physical Therapy Modalities")

AND

2. (MM "Child+") OR "child" OR (MM "Adolescent") OR "adolescents" OR (MM "Youth Sports") OR "youth"

AND

3. (MM "Qualitative Research+") OR (MM "Focus Groups") OR "qualitative"

= 127 Screened for population of young person that included MSK pain/disorder/disease.

Relevant articles – 3 new

1. Houx et al. (2021) No pain, no gain? Children with cerebral palsy and their experience with physiotherapy. *Ann Phys Rehabil Med*, May;64(3):101448. doi: 10.1016/j.rehab.2020.10.002.
2. Crom et al. (2020) Between the lines: A qualitative phenomenological analysis of the theurapeutic alliance in paediatric physical therapy. *Phys Occup Ther Pediatr,* 40(1):1-14. doi:10.1080/01942638.2019.1610138.
3. Paterno et al. (2019) Patient and Parent Perceptions of Rehabilitation Factors That Influence Outcomes After Anterior Cruciate Ligament Reconstruction and Clearance to Return to Sport in Adolescents and Young Adults. *J Orthop Sports Phys Ther.* Aug;49(8):576-583. doi: 10.2519/jospt.2019.8608.
4. DUPLICATE: Ahlqwist and Sällfors C. 2012. Experiences of low back pain in adolescents in relation to physiotherapy intervention. *Int J Qual Stud Health Well-being*, 7:1 15471, DOI:10.3402/qhw.v7i0.15471

Excluded from title

1. Not MSK related pain
   van der Veer et al. 2024; Hurd et al. 2024; Bertoni et al. 2024; Sol et al. 2023; Scholefield et al. 2023; Oyake et al. 2023; Andrews et al 2023; Demeke et al. 2023; an der Veer 2023; Musselman et al. 2023; Caserta et al. 2022; Aviram et al 2022; Cardenas et al. 2022; Livingstone et al. 2022; Feldner et al 2022; Farjoun et al. 2022; Fazzi et al. 2021; Truong et al 2021; Senesac et al. 2020; Pérez-de la Cruz et al. 2020; Pottinger et al. 2020; Kenyon et al. 2020; Cameron et al .2020; Akhbari Ziegler et al. 2020; Güeita-Rodríguez et al. 2019; Jarvis et al. 2019
2. Mixed HCP that included physiotherapists
   Abelsson et al. 2024; Talty et al. 2020; Logan et al. 2020;
3. Not treatment experience:
   a. Explored clinical reasoning of PTs- Christensen et al. 2023; Philp et al 2022;
   b. Physiotherapists training Bertoni et al. 2023; Frygner-Holm et al. 2021

    c. Tool evaluation Hadwin et al. 2023; Nixon-Cave et al. 2019
    d. Telehealth - Wittmeier et al. 2022, 4)Extended scope – Bastiaens et al. 2021
4. Commentary only – Maus et al. 2023; Miciak (2020); Kenyon et al. 2019;
5. Protocol- Estebanez-Pérez et al. 2022

Excluded from Abstract

1. Qualitative survey reasons to treat or not to treat with manual therapy – not experience Driehuis et al. 2023;
2. Not MSK – West et al. 2021;
3. Mixed HCP – McKinnon et al. 2021; Schwellnus et al. 2020
4. Design intervention not experience - Frygner-Holm et al 2021;

Excluded after reading paper

1. Type of patient not described therefore unknown if included MSK: Pelckmans et al 2023; Thomaset al 2002;
2. Adult focus -Rossettini G et al. 2020

AMED - The Allied and Complementary Medicine Database

1. qualitative research or qualitative study or qualitative methods or interview or focus group

AND

2. children or adolescents or youth or child or teenager

AND

3. chronic musculoskeletal pain OR complex regional pain syndrome OR severs disease or calcaneal apophysitis OR osgood-schlatter disease OR knee pain or knee injury or patellofemoral pain OR low back pain or lumbar pain or lumbar spine pain or non specific low back pain OR
   musculoskeletal disorders or musculoskeletal pain or musculoskeletal injuries.

AND

4. physical intervention or physiotherapy or rehabilitation OR exercise therapy OR physical therapy
5.

Year of publication NOT limited – 1995 to current.
= 21

Relevant- 1 new

1. Kuenze et al, 2022. Adolescent patient, parent, and clinician perceptions of rehabilitation after anterior cruciate ligament reconstruction: A **qualitative study.** *J Athl Train.* 2022 Sep 1;57(9-10):929-936. doi: 10.4085/1062-6050-0491.21.

Excluded from title

1. Population-Adult
   Chen 2018; Sheraton 2018; Ezzat 2018; Ôsterberg 2013; Haugland 2012
2. Not physio experience- Burland 2018, Tjong 2014 (return to sport); Macdonald 2017 (validation questionnaire); Chahal 2014; Boonstra 2000; Dixon 1999; Engelbert 1998; Young 1995
3. Not physio Rx- Tabard-Fougere 2016; Cook 2013;
4. Not Qual- Bell 2014; Rathleff 2013; Vieira de Castro 2013; Legg 2008

Excluded from abstract

Activity not physiotherapy - Truong et al, 2022;

Cochrane database

Search child AND pain AND (title abstract and keyword) = 338
333 excluded from title.

Excluded after reading abstract/article:

1. Qualitative but did not specifically look at physiotherapy treatment: France et al. 2023 A meta-ethnography of how children and young people with chronic non-cancer pain and their families experience and understand their condition, pain services, and treatments

RCTS (quantitative only)

1. Leite et al. 2023, Physical activity and education about physical activity for chronic musculoskeletal pain in children and adolescents
2. Takken 2008 Exercise therapy in juvenile idiopathic arthritis
3. Ospina et al. 2021 Physical therapy interventions, other than general physical exercise interventions, in children and adolescents before, during and following treatment for cancer
4. Smith et al. 2023 Surgical versus non-surgical interventions for treating patellar dislocation.

Articles read in full

1. Ahlqwist and Sällfors C. 2012. Experiences of low back pain in adolescents in relation to physiotherapy intervention. International journal of **qualitative** studies on health and well-being [Int J Qual Stud Health Well-being] 2012; Vol. 7. *Date of Electronic Publication:* 2012 Jun 18.
2. Astley et al. 2021. Home-based exercise program for adolescents with juvenile dermatomyositis quarantined during COVID-19 pandemic: a mixed methods study. Pediatric rheumatology online journal. 2021;19(1):159.
3. Birt et al. 2014. Adherence to home physiotherapy treatment in children and young people with joint hypermobility: a qualitative report of family perspectives on acceptability and efficacy. Musculoskeletal care. 2014;12(1):56-61.
4. Blanco-Morales et al. (2020) Implementation of a Classroom Program of Physiotherapy among Spanish Adolescents with Back Pain: A Collaborative Study. International journal of environmental research and public health. 2020;17(13).
5. Crom et al. 2020. Between the Lines: A Qualitative Phenomenological Analysis of the Therapeutic Alliance in Pediatric Physical Therapy. Physical & occupational therapy in pediatrics. 2020;40(1):1-14.
6. Distanti et al. 2018. Perceptions of Rehabilitation and Return to Sport Among High School Athletes With Anterior Cruciate Ligament Reconstruction: A Qualitative Research Study. The Journal of orthopaedic and sports physical therapy. 2018;48(12):951-9.
7. Houx et al. (2021) No pain, no gain? Children with cerebral palsy and their experience with physiotherapy. Annals of physical and rehabilitation medicine. 2021;64(3):101448.
8. Kuenze et al. 2022. Adolescent Patient, Parent, and Clinician Perceptions of Rehabilitation After Anterior Cruciate Ligament Reconstruction: A Qualitative Study. Journal of athletic training. 2022;57(9-10):929-36.

9. Paterno et al. 2019. Patient and Parent Perceptions of Rehabilitation Factors That Influence Outcomes After Anterior Cruciate Ligament Reconstruction and Clearance to Return to Sport in Adolescents and Young Adults. The Journal of orthopaedic and sports physical therapy. 2019;49(8):576-83.
10. Sieczkowska et al. 2022. A home-based exercise program during COVID-19 pandemic: Perceptions and acceptability of juvenile systemic lupus erythematosus and juvenile idiopathic arthritis adolescents. Lupus. 2022;31(4):443-56.
11. Sims-Gould et al. 2018. "I just want to get better": experiences of children and youth with juvenile idiopathic arthritis in a home-based exercise intervention. Pediatric rheumatology online journal. 2018;16(1):59.
12. Williams et al. 2015. Active Treatment for Idiopathic Adolescent Scoliosis (ACTIvATeS): a feasibility study. Health technology assessment (Winchester, England). 2015;19(55):1-242.

Excluded as physical intervention not delivered by physiotherapist.

1. Sieczkowska et al. 2022. A home-based exercise program during COVID-19 pandemic: Perceptions and acceptability of juvenile systemic lupus erythematosus and juvenile idiopathic arthritis adolescents. Lupus. 2022;31(4):443-56.
2. Sims-Gould et al. 2018. "I just want to get better": experiences of children and youth with juvenile idiopathic arthritis in a home-based exercise intervention. Pediatric rheumatology online journal. 2018;16(1):59.
3. Astley et al. 2021. Home-based exercise program for adolescents with juvenile dermatomyositis quarantined during COVID-19 pandemic: a mixed methods study. Pediatric rheumatology online journal. 2021;19(1):159.

= 9 articles included in scoping review.
