# Supplementary material for: Understanding the importance of therapeutic alliance during physiotherapy treatment for musculoskeletal pain in children: a scoping review
Source: Front Pain Res (Lausanne). 2024 Sep 25;5:1452771. doi: 10.3389/fpain.2024.1452771 (PMC11461466; doi:10.3389/fpain.2024.1452771)
Supplement: Supplementary File S2 — Data Extraction Table. [file Datasheet2.pdf]

Finding resilience in me through therapeutic alliance

Study	Data related to the treatment experience from child, parent or physiotherapist perspective.	Are aspects/components of therapeutic alliance discussed in terms of:<br>a) relationship with physiotherapist;<br>b) agreement on management;<br>c) agreement on goals<br>d) other components	Any perceived impact/ change in response to a component of therapeutic alliance
Ahquist and Sallfors 2012	**Perspective of children**<br>• Prior to intervention, children experiencing low back pain described a loss of control over their bodies, their daily routine and ability to govern their daily lives. Identity crisis and reduced self-esteem.<br>• Physiotherapy formed basis for process of change /a reorientation process. Gaining body confidence in daily life.<br>• Children gradually assumed greater responsibility for their own treatment and were ready to take the step to fend for themselves. A process that contributed to greater degree of autonomy.	a) **Perspective of children (relationship).** Physiotherapist motivated and inspired young people to take a more active role and influence own situation.	b) **Perspective of children.** Exercises (management) provided young people the opportunity to have structure and control over their lives (physically and psychologically): 1) focus on treatment shifts focus away from symptoms; 2) increased confidence in their capacity; 3) knowledge reduced fear; 4) Symptoms decline or become less important. Feeling of hope and regaining control over their lives.
Birt et al. 2014	**Perspective of child and parent.**<br>• Intensive exposure to physiotherapy appeared to have empowered parents and children: they gained knowledge, skills and the confidence to adapt exercise and self-manage symptoms.		b) **Perspective of child.** Exercises (management) *'I keep doing the exercises because if they've helped me now and I don't do them then I'm going to start going downhill again'. (Boy 12).* Gaining control and self-management of symptoms.
Blanco-Morales et al. 2020	**Perspective of child**<br>• Quotes reaffirm their new understanding regarding postural self-analysis and the improvement of body awareness.<br>• Children highlighted that the workshops provided a feeling of overall relaxation. They learnt tools to help relax their muscles and stretches that could ease back pain.<br>• Some children revealed that they now associated the workshops with an improvement in their state of health and wellbeing. "*I know how to care for my body a little bit better, if something hurts in one part of the body, now I know "I know how to care for my body a little bit better, if something hurts in one part of the body, now I know how to try*		

	and make it be better, how to cure it, all in all, it's done me good."		
Crom et al. 2020	**Perspective of parents:** Parents felt too underpowered to be able to negotiate about the treatment. They experienced a loss of control over the situation which detracted from their position as parents.		
Distanti et al. 2018	**Perspective of child.** Barriers to returning to physical activity (barriers to recovery) included the social barrier of role adjustment. Children transitioned from a thriving, physically active child to someone who is injured (loss of identity and control/structure of lives). These psychosocial factors appear to have critical importance during recovery transition points.		**c) Perspective of child (goals).** Children were more likely to see their recovery positively if they viewed the process as "overcoming adversity", an optimistic attitude where they believed their experience made them stronger as people overall.
Houx et al. 2021		a) Relationship<br>**Perspective of child.** Easy discussions with physiotherapists (a good relationship) distracted most children from pain.<br>b) Management<br>**Perspective of child.** Most children believed physiotherapy was important (provided clinical benefit in terms of range of motion and functional ability) and therefore, they felt they had no choice but to attend. Stretching was most commonly associated with pain and was perceived as something to be endured for their own good.	**Perspective of child.** Children perceived they had no choice but to attend physiotherapy, quotes portrayed fear of getting worse without physiotherapy *"without physio we wouldn't do anything…if you stay like that you will just get worse."*
Kuenze et al. 2022			**Perspective of child, parent and physiotherapist (a) relationship and (c) goals.** Communication with surgeon via email or messaging application about complications, goal setting and modifying restrictions would enhance confidence through shared understanding of short-term and long-term expectations.
Paterno et al.		**Perspective of child and parent (a) relationship:** Fear and lack of confidence of children were common. The role of the physical therapist as a booster of confidence appeared to be prominent through the different stages of rehabilitation but particularly returning to high level sport without reinjury. Motivation was often described as being regulated by confidence and accountability.	

Williams et al. 2015	**Perspective of child**<br>• Two of the three girls with pain noticed a marked improvement in pain. "*If I stop doing the exercises then I am in a lot more pain and then I will start doing them again. It becomes easier. The exercises themselves they don't really get any easier they just, when I am not doing them I am getting a lot of pain". (Girl)*<br>• Young people reported important outcomes were body awareness/control. *'Giving me more awareness…looking in the mirror and seeing my curve' showing how posture can change how stand which was 'a big improvement'*<br>**Perspective of parents.**<br>Parents described positive outcomes included 'a growth point for my child'. Growth point – about making choices and how these shape you – process had been an education.<br>**Perspective of physiotherapists.**<br>Physiotherapists describe the sense of control that the physical intervention brings. The girls were now doing something positive to help themselves, rather than 'waiting' passively for what may or may not occur. This gave the girls a sense of responsibility and control over their condition. Physiotherapists also described improvements in posture and the effects of this irrespective of possible improvement in the curve. This could give a sense of confidence for the future.<br>*Body confidence – "She was just really, really pleased with it and she actually went out in a vest top last week. For me that was really lovely". Physio.*	**Perspective of child and parent c) Goals.**<br>Families motivations for being the trial included learning to manage a long-term condition.	**(b) management**<br>**Perspective of child, parents, and physiotherapist**<br>Self-management identified as an important consideration across all stakeholders especially in terms of pain, sense of control and confidence.<br>**Perspective of parents.**<br>Parents described the intervention as providing a tool for the future, specifically to help to control pain, to enable my child to adjust her posture and to encourage her to become more physically active to achieve positive health as an adult.<br>"*I think before, if you've never been into a gym you're scared about gym stuff aren't you, but now she's done a few things in the gym and she knows what she's doing . . . and actually she doesn't have to have somebody showing her what to do".(Mother)*

<u>Theme 1: A trusted guide through the ups and the downs</u>

Study	Data related to the treatment experience from child, parent or physiotherapist perspective.	Are aspects/components of therapeutic alliance discussed in terms of:<br>a) relationship with physiotherapist;<br>b) agreement on management;<br>c) agreement on goals<br>d) other components	Any perceived impact/ change in response to a component of therapeutic alliance

Ahquist and Sallfors 2012	**Perspective of child.** 'Coaching from physiotherapist' was a major category. It was one of the ways adolescents mobilised their own resources and was of major importance in the process of change during physiotherapy treatment.	**Perspective of child.**<br>**a) Relationship**<br>• Trust in the physiotherapist is crucial. Children appreciate attitude and professionalism of physiotherapist "warm and open". Importance of someone wanting to help.<br>*"But I think that's exactly what it was, a positive experience. That it was even available, that there was a place where you could go to see someone who was trained and could help you. And then to be able to agree on what the problem is and what to do about it. And even in some way, it wasn't just up to me, but like a joint effort. Yeah, and like someone who cared".*<br>• Physiotherapist was a significant motivator and inspired children to do things they used to and new things for future.<br>b) **Relationship/management**<br>Children expressed inability to manage their training on their own, initially dependant on physiotherapist. With support of physiotherapist children tested, which involved daring to exceed an invisible boundary and challenging uncertainty.	**Perspective of child.**<br>**a) Relationship**<br>• Coaching from physiotherapist and trust strongly contributes to gaining control of back pain and it's consequences.<br>• Physiotherapy served the purpose of secondary control by taking responsibility for adolescents and their treatment through coaching at the start.<br>• Described relief, peace of mind and trust when physiotherapist takes them seriously, listens, tries to understand, explains why the pain feels as it does, and proposes treatment. Affirmation serves as a catalyst. It helps to inspire faith that things will improve.
Birt et al. 2014	**Perspective of parents.** Parents advocated exercises with health professionals present 'a monitored session'		
Blanco-Morales et al. 2020	**Perspective of child.** Children valued physiotherapists (experts) teaching them 'tools' (relaxation, stretches, posture awareness) to self-manage their back pain. Relevant/appropriate education from physiotherapists allowed children to self-analyse own posture and improve body awareness, *"I think about it, before sitting poorly, I correct myself"*		
Crom et al. 2020	**Perspective of parents.** Some parents expressed they wanted transparency in communication with physiotherapists when it came to discussing problems.	• **Relationship**<br>The importance of having trust in the healthcare professional was a key finding of this study.<br>**Perspective of child and parents – TRUST**<br>**Perspective of child and parents.**<br>• Two types of trust were described by children and parents: trust in relational skills and trust in technical skills of the physical therapist.	

		• Parents and children considered the relational skills of the physical therapist to be more important than technical skills.<br>• Trust was pivotal during negotiations about treatment goals and played a key role in their perception of family-centered care.<br>• Children and parents emphasized their need for trust in the therapist to be able to feel safe. This need to feel safe appeared to be a prerequisite for treatment. *“he will not immediately trust the therapist fully; trust grows over time” (*parent)<br>**Perspective of Physiotherapists.**<br>• Physiotherapists stressed their wish to be perceived as a good professional, which they equated more with technical skills than relational trust.<br>• Physiotherapists emphasized their willingness to collaborate with child and parents and acknowledged the importance of trust in the therapist as a prerequisite to strengthen the collaboration between them.<br>• When physiotherapists noticed a problem in the therapeutic relationship, they neither reflected on this problem nor discussed the issue openly with the children and parents<br>• In contrast to parents, physiotherapists did not mention the theme of negotiation. They did not seem, or seemed only slightly, aware of the concerns about relational inequality in the treatment as perceived by children and their parents.<br>**Perspective of parents.**<br>• Parents placed more emphasis on the importance of relational trust: communicating and connecting at the level of the child, transparent in their communication, and offering positive feedback to the child. They also stressed the importance of an open, emphatic, and welcoming attitude of the physiotherapist toward the child.<br>• The parental need for negotiation seemed to be evoked by dependency on the therapist’s	

		qualities and also by a perceived lack of control.<br>**Perspective of children, parents and physiotherapists**<br>Each stakeholder has different tasks and responsibilities within the context of family-centered care and the therapeutic alliance. The therapist brings communication skills, professional skills, and knowledge into the alliance, together with a perspective on the health condition and treatment of the child. The child brings a care need that is situated in a certain context and is among others shaped by child's personal characteristics. Finally, the parents, as experts on their child, bring their strengths, needs, and values into the alliance.	
Distanti et al. 2018	**Perspective of child.** Children struggled with uncertainty about their progress throughout their rehabilitation because recovery at times was non-linear with day-to-day improvements not always evident. These mental challenges contributed to lack motivation to engage in physiotherapy.	A) Relationship<br>**Perspective of child.** A trusting relationship with the physical therapist was a positive recovery factor.<br>a) Management<br>**Perspective of child.** Insufficient guidance and attention from physiotherapist was a negative recovery factor.	**Perspective of child.** Children were more likely to report a negative experience of physiotherapy when they perceived insufficient attention and guidance from their physiotherapist.
Houx et al. 2021	**Perspective of child.** One third of young people reported the physiotherapist continued the exercise regardless of pain and did not listen to them	a) Relationship<br>**Perspective of child.** Most agreed it was important to have a good relationship with the therapist. A sense of betrayal was sometimes apparent: a few children felt that their therapist took advantage when they spoke to them and relaxed to "pull harder" during stretches and a few felt if they complained their therapist made them work harder.	A) Relationship<br>**Perspective of child.** A good relationship with their physiotherapist meant children could have easy discussions which distracted them from pain (however 2 participants felt a good relationship did not affect pain).
Kuenze et al. 2022	**Perspective of child, parents, physiotherapists.**<br>Inconsistencies between surgeon, and physiotherapist source of frustration for young person, parent, and physiotherapist.<br>**Perspective of child and parents.**<br>• Agreed external motivation was helpful to remained disciplined.<br>• Largely unaware of mental toll injury and rehab would take on them both. That the psychological challenges were not stressed enough by their physiotherapist in the early stages of rehab. Most common- emotional	A) Relationship<br>**Perspective of parents and physiotherapists.** consistently depicted their involvement as motivating and supportive.	a) Relationship<br>Interactions with the treating physiotherapist had the potential to both positively and negatively affect perceptions of the rehabilitation process.<br>**Perspective of child**<br>When communication and expectations were consistent children reported better progression in rehabilitation.<br>**Perspective of physiotherapist**<br>• Physiotherapists reported perceived discrepancies between them and surgeons regarding speed of rehabilitation due to lack of

	response to injury *"mentally…I was not prepared"*		communication that undermined healthcare decisions made by both providers.<br>• Some physiotherapists perceived if a parent attended too many rehabilitation sessions while holding unrealistic expectations for their child's recovery, that could be harmful (not expressed by parents or young people)
Paterno et al. 2019	**Perspective of parents.** Physiotherapist *"very important voice in the decision of care of the patients" (mother).* Coordinates different stakeholders , insurance, athletic trainer, surgeon, patient, parents.	a) Relationship<br>**Perspective of child and parents**<br>Fear and lack of confidence were common in children. Role of the physical therapist as a booster of confidence appeared to be prominent through the different stages of rehabilitation but particularly in final stage. Young people and parents described the final stage as most challenging in terms of fear of return to high level activity without reinjury.	
Williams et al. 2015		(a) Relationship.<br>**Perspective of parent**<br>*"I would be disappointed if my child was seeing a physiotherapist who . . . isn't thinking about my child's life in term of education and impacts on how my child feels about themselves. I'd have an expectation of an expert physiotherapist to be incorporating that in their thinking"*	(a) Relationship.<br>**Perspective of child.** Children noted that 'someone checking up on me' (parent or physiotherapist) was an incentive to keep going. (Motivation)

Theme 2: Having a route map

Study	Data related to the treatment experience from children, parents, or physiotherapists.	Are aspects/components of therapeutic alliance discussed in terms of:<br>a) relationship with physiotherapist;<br>b) agreement on management;<br>c) agreement on goals<br>d) other components	Any perceived impact/ change in response to a component of therapeutic alliance
Ahquist and Sallfors 2012	**Perspective of child**<br>• The ways in which children mobilised own resources included 'seeking information.'<br>• Under the category 'process of change', children sought information themselves through the internet and social media and from the school nurse and physiotherapist.	a) Relationship<br>**Perspective of child**<br>• Children sought information from physiotherapist.<br>c) Goals<br>**Perspective of child**<br>• 'Process of Change' -Mutual goal (physiotherapist and child) of body confidence. Collaborative goals part of how progress was monitored.	a) a) Relationship<br>**Perspective of child**<br>When children sought information from their physiotherapist the physiotherapist contributed expertise and the children were given a contextual understanding of their situation. They acquired greater understanding, knowledge, and awareness of their bodies (how their body feels and works) and back problems, that resulted in knowledge on how to proceed. Knowledge

			reduced fear. On the contrary, when young people sought information on social media and the internet, they could not identify themselves within given descriptions (lacking context/ personal narrative). Information was often about adults and made them more worried about the future. Young people report that they are strengthened by finding their resources and that they trust in themselves. They take an active role, assume personal responsibility for feeling better and gain body confidence in daily life. The children attain a new identity and mobilise their resources, realizing that it is possible to feel good even though they still may be in pain. *"So it was like I understood the actual context better because before I didn't really know what that meant and it was a little bit like I didn't dare, but now I just push harder and struggle to make progress".*<br>**b)** Management<br>**Perspective of child.** Focus on treatment shifts focus away from symptoms
Birt et al. 2014	**Perspective of child and parents.** Parents and children gained knowledge about how to manage future exacerbations of hypermobility symptoms.	b) Management<br>**Perspective of child and parents.** Even when the exercise programme reduced symptoms of hypermobility (a shared goal) this did not guarantee adherence to the exercise programme.	
Blanco-Morales et al. 2020	**Perspective of teachers.** The information provided by the physiotherapy activities was powerful. The children were able to learn about and acquire tools for improving and preventing their back pain. Beforehand most children were unaware of how to manage or treat this pain.<br>**Perspective of children.** New knowledge acquired: *"We have learnt knew things so that our back doesn't ache" (FI, PRHS)*- Able to identify ergonomically incorrect postures, self-analyse own posture and improve body awareness. Learnt new stretches that could ease back pain.<br>**Perspective of children and parents.** Reported what was learnt was extrapolated out the classroom.		
Crom et al. 2020		a) Relationship<br>**Perspective of parents and physiotherapists.** Although both physiotherapists and parents	c)Goals<br>A lack of agreement on goals and tasks as well as a lack of therapeutic negotiation may result in

		considered sharing of information to be important, the urgency, quality, and focus of information sharing was different for parents and physiotherapists.<br>**Perspective of physiotherapists.**<br>• An interesting finding was that physical therapists did not share their own wishes regarding the therapeutic alliance with children and parents.<br>• Physiotherapists did not mention the notion of parental responsibility.<br>b) Management<br>**Perspective of parents**<br>Emphasized the importance of parental responsibility for the treatment of their child. They felt responsible for the integral functioning of their child. It seemed parents did not want to, or could not, entirely relinquish responsibility to the therapist. Parents stressed need to receive info from physiotherapist about (small or large) events that occurred during treatment sessions.<br>**Perspective of children**<br>Older children in particular preferred to negotiate about the content of their home exercise programs. However, the negotiations often stalled because the child avoided expressing negative feelings about the treatment. Not feeling comfortable to bring such feelings to the conversation.<br>*"My therapist thinks I do those exercises at home, but very often I forget them…I don't like to do the exercises at home".* (Child 6).<br>**Perspective of physiotherapists**<br>Physiotherapists tended to adhere to what children and parents demanded from them without initiating a discussion. Physiotherapists underlined importance of receiving information from child and parent related to treatment. *"issues, dislikes, or other things that do not fit the ideas of child and parent"* Therapist 9.<br>c) Goals<br>**Perspective of parents.** Some parents mentioned that the physiotherapist focused too much on goals related to motor skills, while they experienced much stress and sorrow due to their child's diagnosis. Stressed the importance of a holistic approach. *"See my child as a whole person and not only focus on my child's problems with motor skills" Parent 6.*	ruptures in the therapeutic alliance and drop-out from treatment.

		Parents wanted to share their own observations at home related to treatment goals with the therapist.<br>**Perspective of children and parents.** Some parents and children stressed the importance of negotiation concerning goals and tasks of the treatment (e.g. about type and scope of home programs). They expected the physiotherapist to determine treatment goals and coordinate the home program, but they also wanted to have a say in this process and discuss the progression of the treatment.	
Distanti et al. 2018	**Perspective of children.** Positive Recovery Factors included: building knowledge about the recovery process and educating children so they possessed knowledge of their specific injury and the recovery process was viewed as beneficial.	b)Management<br>**Perspective of children.** Negative Recovery Factors included over generalised approach to treatment. Standardised and generic exercises seen as too basic and insufficiently motivating as they were perceived as incongruent to the sport-related activity which the young person wanted to return.<br>c)Goals<br>**Perspective of children.**<br>• Highlighted the insufficiency of using physical markers exclusively to evaluate progress during rehabilitation after ACLR.<br>• Participants struggled with uncertainty about their progress throughout their rehabilitation, because recovery at times was non-linear with day-to-day improvements not always evident. These mental challenges contributed to lack motivation to engage in physical therapy.<br>• Positive Recovery Factors included demonstrating individualised goals.	b)Management<br>**Perspective of children.**<br>• More knowledge allowed for a greater sense of control by young people.<br>• Physiotherapists who were readily available to answer any question built participants knowledge and had a positive impact on their experience.<br>• Children who were well informed about the details of their injury and expected timeline of their rehabilitation and were able to anticipate challenges were more likely to maintain a healthy mindset throughout, took a more active role in their rehabilitation and had a greater sense of control.<br>• Uncertainty of progress throughout rehabilitation, activities not linking to their sport (motivating) and rudimentary nature of the exercises led to mental challenges/psychological barriers and lack of motivation to engage in physical therapy.<br>c)Goals<br>**Perspective of children.**<br>• More likely to view rehabilitation positively when goals were self-determined or created in collaboration with physiotherapist.<br>• Individualised considerations for the child’s goals, motivations and personal characteristics aids the child’s perceptions of personal relevance and treatment effectiveness.

Houx et al	**Perspective of children.**<br><br>• The children expressed the perception that many physiotherapists did not understand or did not fully consider their pain.<br>• With regard to therapist-led strategies, some participants reported that they felt their physiotherapist did not understand their pain sufficiently.		c) Management<br><br>**Perspective of children.** It was clear from the interviews that the participants perceived that physiotherapy practices were not universal and that therapists did not all have the same understanding of pain. About one-third of the participants felt their therapists continued the exercise regardless of the pain and did not listen to them.
Paterno et al.		a) Relationship<br>**Perspective of children.**<br>Children described physiotherapists as the resource that filled the gaps of understanding regarding the rehabilitation process. Education and understanding to support motivation. Why I am doing this? Understanding fear, what if I injure again?<br>**b) Goals**<br>**Perspective of children and parents.**<br>Multiple children and parents believed they would have benefited from a more explicit way of knowing how they were progressing in terms of rehabilitation milestones, one quote suggests a *“chart of where I should be…so I could know what I’m shooting for”.* Realistic expectations.	
Kuenze et al. 2022	**Perspective of children and parents. L**ack of young person and parent knowledge presurgery and immediately post was a challenge. All stakeholders perceived a lack of patient and parent knowledge at the time of injury and lack of quality educational resources developed specifically for children were significant barriers to shared understanding of physiotherapist, parents, and young people.<br>**Perspective of physiotherapists.** Importance of access to parent and surgeon was important to facilitate consistency in knowledge and expectation of rehabilitation process.<br>**Perspective of children and parents.** Identified postinjury assumption rehabilitation would be easy due to patients age and prior level of athletic ability. – *“not knowing” (uncertainty)*		b)Management<br>**Perspective of children and parents.**<br>• Lack of knowledge and understanding of rehabilitation timelines, led to feelings of anxiety, frustration and isolation.<br>• Lack of high quality educational resources available to understand recovery process from injury to return to sport, resulted in dependence on orthopaedic surgeon with varied success.<br>**Perspective of children and parents.**<br>• Communication with surgeon via email or messaging application about complications, goal setting and modifying restrictions would enhance confidence through shared

	Largely unaware of mental toll injury and rehabilitation would take on them both. Felt the psychological challenges were not stressed enough by their physiotherapist in the early stages of rehabilitation.		understanding of short-term and long-term expectations.<br>c) Goals<br>**Perspective of children.** Reported finding sources of intrinsic motivation through independent or physiotherapist led goal setting that was helpful in sustaining interest in and commitment to rehabilitation.
Williams et al. 2015	**Perspective of children.** Children reported important outcomes included becoming more informed and aware of posture.<br>**Perspective of children and parents.** Children and parents had unmet information needs, They wanted to find out what it has been like for other people rather than worst case scenarios on the internet. Information with genuine input from children and parents.	c)Goals<br>**Perspective of children and parents** Parents and children did not remember much about goal-setting.. One child described goal-setting as useful because *'I could see what I needed to focus on rather than just that I have bad back pain'.*	c)Goals<br>**Perspective of physiotherapists.** Although goals were described as a useful way of giving children a sense of control, given that children with idiopathic scoliosis are generally not restricted functionally, fit and well and may have no pain, physiotherapists described the challenge of setting goals:<br>*I think as physios we're used to people coming in and pain being their primary problem and this is completely different. It's nice in that way . . . to be treating someone who . . . you're trying to make them better.*

Theme 3: Take me seriously but make it fun

Study	Data related to the treatment experience from children, parents and physiotherapists.	Are aspects/components of therapeutic alliance discussed in terms of:<br>a) relationship with physiotherapist;<br>b) agreement on management;<br>c) agreement on goals<br>d) other components	Any perceived impact/ change in response to a component of therapeutic alliance
Ahquist and Sallfors 2012		*c) Management*<br>**Perspective of children. "***I've found that it has become more and more fun to do things so I've been motivated all along. And now when I can see progress, I feel like, wow! I used to play tennis and I want to start doing that again and I want to dance again too".*	A) Relationship<br>**Perspective of children.**<br>• Children reported **immense sense of relief** when they see the physiotherapist and pain and problems taken seriously.<br>b)Management<br>**Perspective of children.**<br>• Children are able to divert their thoughts and forget the pain when they are enjoying themselves (reduced pain and increased energy). *"They feel the joy of freedom without feeling pain"*

			• Exercise and treatment made young people feel good – do their school work, see friends and have fun.
Birt et al. 2014	**Perspective of parents**<br>• Older young people expected to take responsibility for completing exercises. A few parents discussed tension when the young person did not meet expectation and did not complete exercises, or it became a *"bone of contention in the house"* (Mother 24).<br>• A couple of parents described the exercises as *"monotonous"* (Mother 13)<br>• Exercises were observed to improve quality of life more broadly for some children *"mentally she's a lot happier" (Mother 20).*<br>**Perspective of children**<br>Older young people in secondary education were less motivated to do exercises. *"…if I was bored and had nothing to do I'd do something like the exercises"* (Girl 22).	• Management<br>**Perspective of children and parents.**<br>• Importance of exercises being fun and fitting in with normal routines.<br>• Doing exercises with others (siblings and parents) as the exercise turned into a competition e.g. *"who can balance the longest"* (Father 5). Consideration of wider family network.<br>**Perspective of children**<br>• Children found the hand exercises the easiest to complete and found them fun to do. They were easy to fit into a normal routine as they could be done in the car or watching television.	
Blanco-Morales et al. 2020	**Perspective of children.** *"It's really fun, and also we have learnt new things…"* (Child)		
Crom et al. 2020		a) Relationship<br>**Perspective of children.** Children did express a need for feeling safe with regard to contact and communication, they wanted to experience a relaxed atmosphere with room for jokes. Children wanted the therapist to smile a lot and be joyful, but at the same time, they wanted the therapist to be respectful.*"making jokes is funny, but only at the right moment" Child 4.*	
Houx et al. 2021	**Perspective of children** Most children did not enjoy physiotherapy sessions. Sessions described as boring and tiring.		**Perspective of children.** The experience of musculoskeletal pain during stretches led them to a dislike of physiotherapy.
Kuenze et al. 2022	**Perspective of child and parent.** Several children and parents indicated a network of support going through similar experiences would be helpful *"I wish there was a program…that you just go and talk"* (child). Need for holistic support with a child's mental health taken seriously.		

	"…Nobody tells you about the head game" (Parent).		
Paterno et al. 2019	**Perspective of child and parent.** The middle period of rehabilitation was described as a period of frustration and depression, yet physiotherapists could keep children motivated. "*He (physiotherapist) was the person I loved to talk to no matter what. We talked about music, sports…we just have a good old time" (child).*		
Williams et al. 2015	**Perspective of parents**<br>Some parents described how their children enjoyed the exercises.	a) Relationship<br>**Perspective of children**<br>Children described a positive relationship with the physiotherapists that was both interesting and fun.	
